# Draft Genome Sequence of *Lysinibacillus fusiformis* Strain SW-B9, a Novel Strain for Biotransformation of Isoeugenol to Vanillin

**DOI:** 10.1128/genomeA.00289-15

**Published:** 2015-04-16

**Authors:** Liqing Zhao, Guanhui Bao, Beibei Geng, Jiangning Song, Yin Li

**Affiliations:** a Department of Food Science and Engineering, College of Chemistry and Chemical Engineering, Shenzhen University, Shenzhen, Guangdong, China; b School of Bioscience and Bioengineering, South China University of Technology, Guangzhou, Guangdong, China; c CAS Key Laboratory of Microbial Physiological and Metabolic Engineering, Institute of Microbiology, Chinese Academy of Sciences, Beijing, China; d National Engineering Laboratory for Industrial Enzymes and Key Laboratory of Systems Microbial Biotechnology, Tianjin Institute of Industrial Biotechnology, Chinese Academy of Sciences, Tianjin, China; e Department of Biochemistry and Molecular Biology, Faculty of Medicine, Monash University, Melbourne, Australia

## Abstract

*Lysinibacillus fusiformis* SW-B9 was the first reported strain in *L. fusiformis* showing effective biotransformation of isoeugenol to vanillin. Here, we report the annotated genome of strain SW-B9, which has special pathways for producing vanillin. The genome will provide a genetic basis for better understanding the physiology of this species.

## GENOME ANNOUNCEMENT

*Lysinibacillus fusiformis*, which prior to 2007 was known as *Bacillus fusiformis*, is a Gram-positive nonmotile bacterium of the genus *Lysinibacillus* (1). The majority of studies on *L. fusiformis* are related to its pathogenicity (2, 3), and few researchers have focused on its industrial and agricultural roles. We previously isolated *L. fusiformis* SW-B9 from soil, which can produce high amounts of vanillin from isoeugenol (4). Vanillin is widely used in the beverage, food, pharmaceutical, and medical industries (5). Several strains have been shown to be able to convert eugenol or isoeugenol to vanillin (5, 6). However, for *L. fusiformis*, only the SW-B9 strain was reported for its biotransformation of isoeugenol to vanillin (4). Therefore, the complete sequencing of strain SW-B9 will not only enrich the genome sequence database of *L. fusiformis* but also further our understanding of the genetic, phylogenetic, and physiological properties of this strain.

The genomic DNA of strain SW-B9 was sequenced using the MiSeq system. The whole-genome shotgun run yielded 2,430,573 paired-end reads, accounting for 1,220,147,646 bases in total. *De novo* assembly was performed using SPAdes (7), resulting in an assembly of 22 contigs of >1,000 bp. The total size of the assembly was 4.7 Mbp, with an *N*_50_ of 997.350 kbp and a G+C content of 37%. The annotation of the genome was accomplished with the NCBI Prokaryotic Genome Automatic Annotation Pipeline (PGAAP) (8). A total of 4,400 coding DNA sequences, 70 tRNAs, and 28 rRNAs were predicted. Out of all the genes, 1,721 coding sequences (CDSs) were assigned to 178 KEGG pathways.

A comparative genome analysis was used to measure the similarities between strain SW-B9 and other strains using the reciprocal smallest distance (RSD) (9). A total of 4,040 CDSs, accounting for 92% of the coding genes in the SW-B9 strain, are shared with *L. fusiformis* RB-21 (GenBank accession no. JPEF00000000.1). These results indicate that most of the coding sequences in the SW-B9 strain are highly conserved within other *L. fusiformis* strains, although there is no vanillin biotransformation in these strains. Monooxygenases were considered to be the enzymes that convert isoeugenol to vanillin (10). To find the isoeugenol monooxygenase gene (*iem*), we used the *iem* genes reported in *Pseudomonas putida* IE27 (11), *Pseudomonas nitroreducens* Jin1 (12), *Neofusicoccum parvum* isolate UCR-NP2 (13), *Verticillium alfalfae* VaMs.102, and *Colletotrichum fioriniae* PJ7 (14), to blast against all proteins in the SW-B9 strain. The results show that there is no similar gene in strain SW-B9. However, in the genome of strain SW-B9, we found 15 candidate monooxygenases, enabling an exploration of the mechanism of vanillin conversion in this strain. Therefore, we speculate that the pathway to produce vanillin in SW-B9 is special, and further genetic research will be worthwhile.

The genome sequence of strain SW-B9 serves as a useful resource for further investigation of the molecular basis of its potential in the biotransformation from isoeugenol to vanillin. Systematic annotations of the genome will reveal physiological differences among the various *L. fusiformis* strains.

### Nucleotide sequence accession numbers. 

This whole-genome shotgun project has been deposited at DDBJ/EMBL/GenBank under the accession no. JRBA00000000. The version described in this article is the first version, JRBA01000000.
